# Microwave Synthetic Routes for Shape-Controlled Catalyst Nanoparticles and Nanocomposites

**DOI:** 10.3390/molecules26123647

**Published:** 2021-06-15

**Authors:** Clare Davis-Wheeler Chin, LaRico J. Treadwell, John B. Wiley

**Affiliations:** 1 Department of Chemistry and Advanced Materials Research Institute, University of New Orleans, New Orleans, LA 70148, USA; cdavisw@sandia.gov; 2 Advanced Materials Laboratory, Sandia National Laboratories, 1001 University Blvd. SE, Suite 100, Albuquerque, NM 87106, USA; ljtread@sandia.gov

**Keywords:** microwave processing, nanoparticles, nanotubes, nanocomposites

## Abstract

The use of microwave irradiation for the synthesis of inorganic nanomaterials has recently become a widespread area of research that continues to expand in scope and specialization. The growing demand for nanoscale materials with composition and morphology tailored to specific applications requires the development of facile, repeatable, and scalable synthetic routes that offer a high degree of control over the reaction environment. Microwave irradiation provides unique advantages for developing such routes through its direct interaction with active reaction species, which promotes homogeneous heat distribution, increased reaction rates, greater product quality and yield, and use of mild reaction conditions. Many catalytic nanomaterials such as noble metal nanoparticles and intricate nanocomposites have very limited synthetic routes due to their extreme temperature sensitivity and difficulty achieving homogeneous growth. This work presents recent advances in the use of MW irradiation methods to produce high-quality nanoscale composites with controlled size, morphology, and architecture.

## 1. Introduction

In the past three decades, the use of microwave (MW) irradiation as a thermal input for chemical reactions has attracted considerable attention across a wide variety of applications, including organic/peptide synthesis, analytical chemistry, organometallics, assembly of catalytic heterostructures, polymer chemistry, drug discovery, and biochemical processes [1,2]. Through direct interaction with active species in MW-transparent reaction vessels, MW irradiation enables homogeneous heat distribution, promotes increased reaction rates and reduced reaction times, enables the use of milder reaction conditions, and produces greater yields of desired products. Despite its widespread use in other fields, MW irradiation has only recently been recognized as a viable method to improve the regulation of NP formation and enhance product quality and yield [3]. Traditional methods achieve synthesis via conductive heating, wherein heat is driven through vessel walls to reach the reaction mixture within [2]. As such, the rate of heat transfer and equilibrium temperature are significantly affected by the thermal conductivity of the vessel materials. The temperature of the vessel will be greater than that of the reaction mixture until thermal equilibrium is reached, which results in a prolonged reaction time with a restricted amount of control over the heating rate and equilibrium reaction temperature. For materials that are sensitive to heating rate, equilibrium temperature value, and temperature stability, this lack of control offered by conductive heating methods can greatly reduce product quality.

In contrast, MW irradiation utilizes “in core” volumetric heating to achieve efficient and uniform heating throughout the volume of the liquid medium via direct coupling of MW energy to MW-active species in the reaction mixtures [4]. Direct coupling produces instantaneous localized superheating of reaction species that respond to the electrical or magnetic components of the incident MW field, promoting even heat distribution and uniform heating throughout the mixture [5]. This leads to more homogeneous nucleation and shorter crystallization times, which are beneficial for forming size- and shape-controlled colloidal NPs. MW irradiation also enhances heating and reaction rates by initiating the rapid transfer of MW energy to reaction species on a timescale of 10^−9^ s, much faster than the 10^−5^ s timescale of kinetic molecular relaxation [2]. The resulting non-equilibrium condition enhances reaction rates and product yields.

The rapid heating provided by MW irradiation has allowed the development of facile synthetic routes that eliminate or greatly mitigate complex, time-intensive processing, high temperature demand, and the use of dangerous nucleation-inducing agents such as metal carbonyls [6,7]. Furthermore, MW conditions have been shown to induce specific interactions between precursors, surfactants, intermediate species, and the incident electromagnetic field; these interactions enable production of NP compositions and morphologies that are not achievable using conventional heating methods [8]. In their review of MW synthesis of inorganic colloidal nanocrystals, Baghbanzadeh et al. suggest that the unique nature and effects of MW dielectric heating should be considered “special MW effects” [8]. This category includes the “superheating” effect observed in solvents at ambient pressure, the selective heating of strongly MW-absorbing heterogeneous catalysts or reagents in less-polar reaction media, and the elimination of barrier effects caused by inverted temperature gradients. Table 1 summarizes the key advantages of MW heating for various applications in nanomaterials synthesis.

## 2. Fundamentals of MW Heating

**Figure 1:**
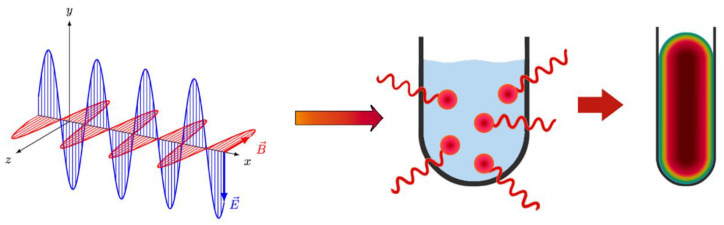
Process of MW heating: microwave radiation interacts directly with the reaction mixture and induces instantaneous localized superheating, which leads to an even heat distribution throughout the mixture.

The heating effects induced by an incident MW electromagnetic field primarily result from the interaction of the electric field component with charged particles in the material, although interaction between the MW magnetic field and the magnetic dipoles of the material can also occur (Figure 1) [19]. Interaction with the MW electric field is dependent upon the mobility of the charged particles in the material, and can give rise to one or both of two major heating processes. Dipolar polarization (*P*_d_) involves the bound dipoles of polar solvent molecules or reagents aligning in the same direction of the incident MW electric field. Rotational motion created by the molecule as it attempts to reorient itself to the oscillating MW electric field results in the transfer of energy. The polarity of the molecules in the reaction mixture and their ability to align with the field determine the coupling ability of this mechanism [17]. The value of *P*_d_ in a material exposed to an electric field with strength *E* is given by Equation (1):

(1) $$P_{d}=\varepsilon_{0}\left(\varepsilon_{r}-1\right)E$$

where *ε*_0_ and *ε*_r_ represent the free space and relative material permittivity, respectively [19].

For materials with higher charge carrier mobility, MW heating effects arise primarily from the movement of charged particles in response to the MW electric field [17]. This process, known as ionic conduction, involves oscillation of the charged particles back and forth under the incident MW electric field and collision with neighboring molecules or atoms. Agitation from these collisions produces heat, the amount of which is a function of the reaction temperature: with increasing temperature, energy transfer from ionic conduction becomes more efficient [2]. Ionic conduction pathways represent a much greater capacity for heat generation compared to heat-generation pathways from dipolar polarization (dipolar rotation). This has a significant impact on synthesis strategies for MW heating, particularly on the choice of solvent for the reaction mixture. 

The ability of a particular reaction species to convert MW energy into heat is expressed as its loss tangent (tan *δ*), which is determined by Equation (2):

(2) $$\tan\;\delta =\frac{\varepsilon^{\prime \prime }}{\varepsilon^{\prime }}$$

in which the dielectric loss *ε*″ (complex permittivity) quantifies the efficiency with which the species converts EM radiation into heat, and the dielectric constant *ε*′ (relative permittivity) represents its polarizability in an electric field. For NP synthesis, selecting tan *δ* is most often applied to solvents, which are classified according to these values as high, medium, or low MW absorbers. A high tan *δ* value is desirable for good absorption of MW radiation and a subsequently high heating efficiency, although non-polar solvents with low tan *δ* can be useful for certain synthetic routes when combined with high-absorbing species [2]. Table 2 contains a list of tan *δ* values for common solvents.

While tan *δ* is useful for describing the interaction of reaction species with the electric component of MW irradiation, analogous concepts are needed to describe interactions with the MW magnetic field. The corresponding magnetic field terms to *ε*′ and *ε*″ are dipole moment or permeability *μ*′, and magnetic loss factor *μ*″, respectively [8]. Considering the wide variety of nanoparticles and nanocomposites that could be produced using MW heating methods, it is important to consider all of these factors. 

## 3. Advances in Commercial MW Design for Research Applications

A significant increase in the production of dedicated MW reactors specifically designed for chemical synthesis provides researchers with a high degree of control and in situ monitoring of reaction temperature, pressure, and even timed addition of reaction species [17]. In microwave reactors designed for materials synthesis, MW radiation with a frequency of 2.45 GHz (λ = 12.25 cm) is produced by magnetron generators with microprocessor-controlled power outputs that respond in real time to maintain target ramp rate and reaction temperatures. Continual advances in software, pressure and temperature monitoring hardware, and the availability of targeted accessories have greatly expanded the efficiency, control, and range of chemistry. This section will provide a brief overview of recent advancements in MW reactor design, applications, and hardware for enhanced reaction control.

### 3.1. Single-Mode Cavity Design for Improved MW Energy Distribution

From early experimentation to current research, a significant barrier to achieving optimal energy absorption and homogeneous heat distribution has been caused by the “multimode” cavity design used by most dedicated synthesis MW reactors [20]. These systems are based upon the manipulation of MW-wavelength radiation by the cavity dimensions of domestic microwave ovens, which were designed to maximize the number of MW field configurations (modes) to expand their versatility for food preparation [9,19,20,21].

In multimode instruments, MW irradiation generated by one or two magnetrons is directed into the cavity using a waveguide and distributed with a mode stirrer. The microwaves interact with the sample after reflecting from the walls of the cavity, creating an uneven heat distribution and leading to the formation of hot and cold spots. Constant rotation of samples during the reaction can partially offset this effect, but lack of repeatability and the inability to calculate delivered power remain serious concerns for chemical applications with multimode MW reactors [11].

**Figure 2:**
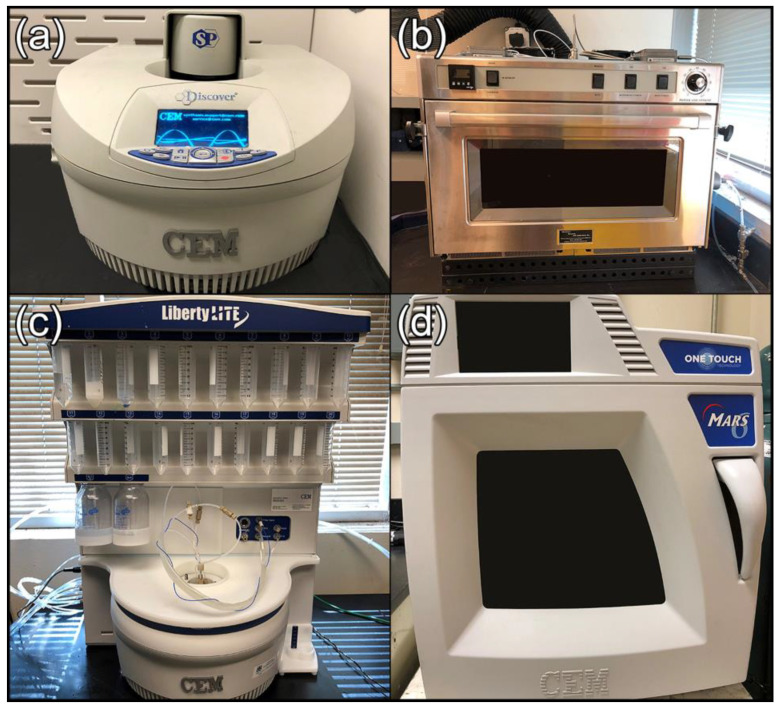
Microwave reactors with advanced design features: (**a**) single-mode cavity CEM Discover SP; (**b**) Microwave Research and Applications BP-210 processing MW with reaction temperatures up to 1800 °C; (**c**) CEM Liberty Blue with critical reagent delivery system; (**d**) CEM MARS6 with high-throughput capacity and fiber optic temperature sensor.

In response to these limitations, many suppliers have developed single-mode benchtop instruments that use a single magnetron to generate a homogeneous energy field with high power intensity [11,19]. Their single-mode cavities produce MW fields with well-defined spatial distribution, allowing sample placement at points where MW radiation with known intensity will be evenly applied. Examples of single-mode benchtop MW reactors include the CEM Discover SP series (Figure 2a) [22], the Biotage Initiator+ [23], and the Anton-Paar Monowave 300 [24]. While single-mode instruments represent a promising advancement for MW energy distribution, factors such as reaction mixture and vessel composition must be carefully controlled to prevent the development of thermal gradients [11].

### 3.2. Expanded MW Capabilities for Chemical Synthesis and Processing

From its initial limitation to extraction, digestion, and organic synthesis reactions, MW chemistry has greatly diversified in scope and specialization. As demonstrated by the systems pictured in Figure 2, commercial offerings over the past several years have dramatically expanded to allow extreme temperature and pressure conditions, use of specialized atmospheres, timed reagent addition, interchangeable reaction vessels, and in situ investigations using specialized reactors [9,10,19,21,25]. The BP-210 Processing MW (Microwave Research Association, Inc.) shown in Figure 2b is a pneumatic system with pressure and vacuum sources that can create extreme reaction environments up to 1800 °C and 2200 psig [26]. The CEM Liberty Blue (Figure 2c) was designed for automatic peptide synthesis but can be used for applications as diverse as nanoparticle synthesis. Its distinctive feature is the Flex-AddTM critical reagent delivery system, which allows reagents to be delivered at the desired quantity and timing during the reaction [27]. The CEM MARS6 shown in Figure 2d offers both high throughput (running up to 16 100 mL vessels at once) and high precision over reaction parameter control via fiber optic temperature and pressure sensors. The removable rotor creates a multifunctional cavity that can easily be used for single vessel and open-top reactions [28].

### 3.3. Advanced Hardware for MW Process Elucidation and Enhanced Reaction Control

While MW heating methods are well established for chemical synthesis and processing, there remains a lack of consensus on which aspects of MW irradiation create the dominant pathways for enhanced reaction yields, use of milder conditions, and accelerated reaction rates [9]. The most contentious issue is whether all these phenomena can be attributed to MW thermal processes (Arrhenius phenomena), or if nonthermal processes such as magnetic field oscillation and solvent interactions also contribute to the unique effects of MW heating [8]. Recent advances in MW hardware may help elucidate these fundamental processes and provide more definitive answers to the current debate. 

#### 3.3.1. Reaction Vessel and Susceptor Materials

**Figure 3:**
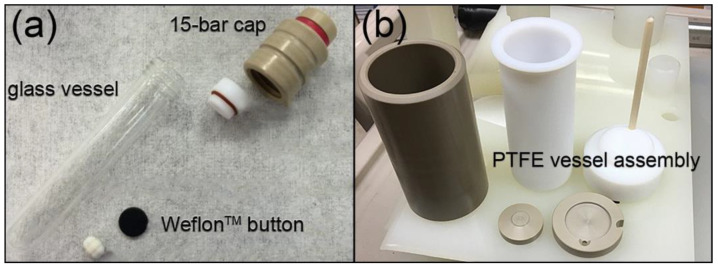
MW-transparent reaction vessel assemblies: (**a**) glass reaction vessel with pressure-responsive cap and graphite-doped Teflon (WeflonTM) button as MW susceptor; (**b**) solvothermal PTFE control vessel with thermowell for fiber optic temperature sensor. All equipment supplied by Milestone Srl.

MW transparency is a critical aspect for reaction vessel design, allowing direct interaction of MW irradiation with the reaction mixture. As MW reactors became specialized for chemical applications, borosilicate glass vessels with pressure-responsive caps became the most common design (Figure 3a). Despite their high MW transparency, borosilicate glass vessels were prone to microfracture development that posed serious safety and equipment hazards. Improvements in design led to the development of engineered safety controls, such as the PTFE vessel and assembly shown in Figure 3b. Silicon carbide (SiC) reaction vessels are another popular replacement for borosilicate glass [20]. The high MW absorptivity of SiC allows the vessels to also act as susceptors, providing faster heating, creating a more uniform temperature distribution throughout the reaction mixture, and decreasing the energy input required to achieve the desired reaction temperature. 

The availability of commercial MW susceptors such as graphite-doped Teflon (Weflon^TM^) that are tailored to chemical synthesis has significantly expanded the range of solvents that can be used in MW synthetic routes. Non-polar solvents, which are normally MW-inactive, can be used in conjunction with susceptors for temperature-sensitive reactions [2]. Polar solvents can act as a heat sink to pull thermal energy away from temperature-sensitive reactants, thus substituting a non-polar solvent, which acts to directly energize reactant molecules. This results in an even temperature increase during heating, homogeneous energy distribution, and steady maintenance of equilibrium temperature (Figure 1) [5].

#### 3.3.2. Fiber Optic Temperature and Pressure Controls

Temperature control and monitoring components have also undergone rapid improvements, most notably through the availability of fiber optic temperature probes as a replacement for the contactless infrared temperature sensors found in most older units [29]. These external IR sensors are integrated into the MW cavity and detect the surface temperature of the reaction vessel from a set distance, often during the rotation of multiple vessels [11]. While the low cost and durability of these sensors was an advantage for early adoption of MW chemistry, their measurements are restricted to the vessel surface and cannot be used to accurately determine the temperature or thermal distribution of the reaction mixture. Previous studies have found significant temperature variations and thermal gradients not represented by the IR sensors [12,30].

In contrast, fiber optic (FO) temperature probes are designed to be immersed directly within the reaction mixture to afford precise temperature monitoring and control. FO probes typically consist of a sensor crystal mounted at the end of a FO cable, and are inserted into protective Teflon-coated ceramic or sapphire thermowells built into the control vessel assembly (see Figure 3b). Readings from the FO sensor are used to adjust the power input during reaction, and have been shown by multiple studies to provide far more precise and accurate temperature control compared to external IR sensors [12]. FO probes can also be used for in situ pressure monitoring.

## 4. Future Directions: Facilitating Scale-Up

### 4.1. Quantitative Computational Modeling

Goyal et al. recently addressed significant gaps in the fundamental understanding of MW reactors affecting scale-up of chemical synthesis and processing for industrial applications [10]. Key factors such as heating uniformity and rate can vary during scale-up via mechanisms that are poorly understood, but can substantially alter operating efficiency and product yield. While existing computational models offer some insight beyond experimental observations, the complexity of EM field distribution within MW reactors permits only qualitative results from these models [30]. Recent advances in commercial software and computational resources offer a promising means of developing quantitative numerical models to expand understanding of fundamental MW processes, provide guidance for advanced reactor design through performance prediction and experimental validation, and elucidate scale-up behavior for expansion of MW synthesis and processing to industrial capacity [25].

Key results of the predictive computational model designed by Goyal et al. included the low MW penetration depth, especially for solvents with high tan δ, and the confinement of heat generation to a narrow surface region when vessel size exceeds penetration depth [10]. Both of these factors severely limit uniform heat distribution throughout the reaction system, which represents a major barrier to scale-up. Development of quantitative computational models and other scale-up techniques is ongoing.

### 4.2. Continuous-Flow MW Reactors

Gawande et al. described the use of a single-mode MW reactor used for continuous flow reactions under high temperature and pressure conditions of 310 °C and 60 bar, respectively [9]. The very low power demand (0.6–6 kW at 2.45 GHz) and facile applicability to both small and large volume systems have made continuous flow processes very promising tools for facilitating scale-up from laboratory to industry level use. Industrial applications would further benefit from other features of flow synthesis, such as the ability to conduct real time monitoring and instantaneous adjustments of reactions in progress. Widespread adoption of continuous flow MW reactors is currently limited by challenging reaction kinetics of the combined reagents during flow, but the topic remains an active area of research.

## 5. Microwave Synthetic Routes to Catalyst Nanomaterials

### 5.1. Synthesis of Colloidal Catalyst Nanocrystals via Conventional Heating Methods

Colloidal metal and metal oxide catalyst nanocrystals (NCs) are generated from the decomposition of metal precursors (including metal–organic and metal–ligand complexes, and metal salts such as nitrates, chlorides, and acetylacetonates) in a liquid medium in the presence of surfactants, soft templates, or coordinating solvents to control particle growth with respect to shape, size, and agglomeration [31]. One common route is wet chemical reduction, in which the selected reaction species are heated to induce precursor decomposition under controlled atmosphere and pressure conditions. Nucleation is then induced through generation of zero-valent metal nuclei, from which growth into NPs proceeds via Ostwald ripening [32]. Several reviews provide extensive guidance for achieving desired size, shape, and composition via wet chemical synthetic routes [14,17,31,32,33,34,35,36,37].

Wet chemical reduction methods require careful selection of reaction species and understanding of their interaction during NP formation. One critical factor for selection relates to maintaining NP dispersibility within the colloid. Metal NPs produced during cluster formation in solution tend to agglomerate, creating large aggregate structures that reduce catalytic activity and decrease flexibility for use in composite nanoarchitectures [38]. Different types of stabilization methods are employed to prevent agglomeration, including stabilization via coordination of sterically demanding organic ligands bound to the NP surface to create physical separation from nearby NPs. In their examination of oleylamine (OAm) as a stabilizing agent for synthesis of colloidal metal NPs, Mazumber et al. found that steric hindrance from the long OAm alkyl chains prevented contact between the high-energy surfaces of the growing Pt NPs and gave products with a very narrow size distribution [39,40].

These synthetic routes employ conductive heating and use heating mantles, hot plates, oil baths, or solvothermal vessels such as Parr bombs. Conductive heating is a slow and inefficient method for thermal energy transfer, limited by dependence on convective currents and the thermal conductivities of various components and materials. Temperature differentials between the reaction vessels and solutions, along with the development of thermal gradients, are key factors that limit size and morphological control and reduce product quality and yield.

### 5.2. MW Heating as an Alternative Route to Shape-Controlled Colloidal Nanocrystals

While wet chemical reduction remains a widely-used and reliable method for many types of colloidal NCs, it is not well suited for the synthesis of size-controlled, faceted noble metal catalyst NPs. Effective control of NP size, shape, and agglomeration by stabilizing agents such as OAm is dependent on the thermal energy within the system. These species must overcome the activation energy barrier for coordination or binding to the NP surface, which requires that sufficient energy is available throughout the reaction mixture. The intrinsic limitations of conductive heating create unavoidable barriers to efficient thermal energy transfer and distribution in the most common wet chemical reduction routes (e.g., solvothermal synthesis in a Parr bomb). This creates a loss of control over NP growth and greatly increases the occurrence of agglomeration that cannot be fully eliminated from conductive heating methods. These methods also lack adequate control over the complex thermodynamic factors and kinetic processes during NP nucleation and growth, including supersaturation, homogeneity of the nucleation event, preferential binding of surfactant species to crystalline facets, and precursor/surfactant ratios.

In response to those challenges, new synthetic routes for faceted, size-controlled catalyst NCs utilize MW heating as an alternative energy input. The ability of MW irradiation to heat a reaction mixture uniformly provides an effective solution to the issues of stabilizing agent activation and agglomeration prevention [41]. Unlike conductive heating, MW heating methods do not have intrinsic limitations on the transfer and distribution of thermal energy. Uniform heat distribution, which can be enhanced using MW susceptors, promotes the interactions between stabilizing agents and NP surfaces that produce the desired size and morphology.

Furthermore, achieving good size and shape control of metallic NPs is highly dependent on controlling reaction temperatures and heating rates that govern particle nucleation and growth. The use of MW-transparent vessels allows MW to penetrate the reaction mixture to give homogenous and simultaneous rapid heating to quickly reduce metal precursors to their zero-valent state, promoting homogeneous nucleation and growth of metallic NPs. Distinct, homogeneous nucleation events and shorter crystallization times are beneficial to the formation of shape-controlled catalyst NPs. In summary, the greater degree of control and precision offered by microwave heating methods creates a highly tunable and repeatable strategy for producing high-quality NPs and other nanocomposites.

### 5.3. Nanoscale Platinum Catalysts

Platinum nanoparticles (Pt NPs) are highly efficient catalysts for photocatalytic water splitting and the redox reactions of oxygen, hydrogen, methanol, and other fuels [42,43,44]. The shape and size of Pt NPs can be controlled to select for specific catalytic reactions, and can also influence interparticle interactions, self-assembly, dielectric properties, and light absorption and scattering [32,45,46]. The synthesis of Pt NPs with controlled size and morphology is important in many areas of catalysis, including the development of alternative energy devices that rely on the efficiency of platinum-catalyzed redox reactions [40,47]. Platinum nanoparticles have been well established as one of the most efficient materials for a diverse array of catalytic applications, including hydrogen reduction and methanol oxidation reactions, performance in polymer electrolyte and direct methanol fuel cells, many types of hydrocarbon hydrogenation/dehydrogenation reactions, and a wide variety of other photovoltaic and photoelectrochemical systems [32,43,48].

Highly dispersible Pt NPs offer increased flexibility for constructing nanoarchitectures for catalysis, expanding the functional possibilities of the NPs beyond deposition onto a substrate or support and facilitating incorporation of the NPs into more diverse constructs. Dispersible Pt NPs have been incorporated into mesoporous oxide and silica matrices via capillary inclusion and nanoparticle encapsulation methods to make highly efficient heterogeneous catalysts [45]. More generally, the high loading selectivity enabled by dispersible Pt NPs is ideal for low and controlled catalyst loadings that significantly impact the durability, cost-effectiveness, and performance of fuel cell designs [44]. A facile synthesis of highly dispersible Pt NPs offers great potential contributions for the design and function of advanced fuel cells, water-splitting architectures, and other heterogeneous catalyst systems.

Despite the widespread use of nanoscale platinum for catalysis, only a few facile synthetic strategies for producing monodispersed Pt NPs have been reported [32,44]. The current benchmark method for the production of small (< 10 nm), monodispersed, and highly crystalline Pt NPs with a high degree of morphological control is an organic phase synthesis first reported by Wang et al. [45]. A crucial element to the success of this method was the injection of Fe(CO)_5_ to control the nucleation and growth rates of the Pt NPs.

MW heating methods for producing high-quality, shape-controlled catalyst NPs have been reported, but remain limited in comparison to metal oxide and chalcogenide NPs [7]. Since it is well documented that the formation of Pt NPs is extremely sensitive to changes in temperature, the greater degree of control offered by microwave heating allows a much greater tunability of both NP size and shape [44,49]. Green MW synthesis of Au, Pt, and Pd NPs using glycerol as solvent and reducing agent was reported by Kou et al. [50]. MW synthesis of Pt NPs has been reported by several other groups, but none have produced highly crystalline or shape-controlled NPs [41,51,52].

### 5.4. MW Synthesis of Catalyst Nanoparticles

Our studies on creating an alternative approach for a safe, facile, and reproducible synthesis found that highly crystalline, shape-controlled, dispersible Pt NPs could be reliably produced using a “one pot” microwave heating method. The method was adapted from the high-temperature organic phase synthesis of Wang et al., which utilized a non-polar solvent to facilitate interactions between Pt^0^ nuclei and the shape-control agent oleylamine (OAm) to produce 10 nm dispersible Pt nanocubes [45]. The key advantage of MW irradiation was its promotion of uniform volumetric heating, which enabled a spontaneous and homogeneous nucleation event. This eliminated the need to induce nucleation using toxic and highly reactive Fe(CO)_5_, and created a facile synthetic pathway to faceted Pt NPs [7].

**Figure 4:**
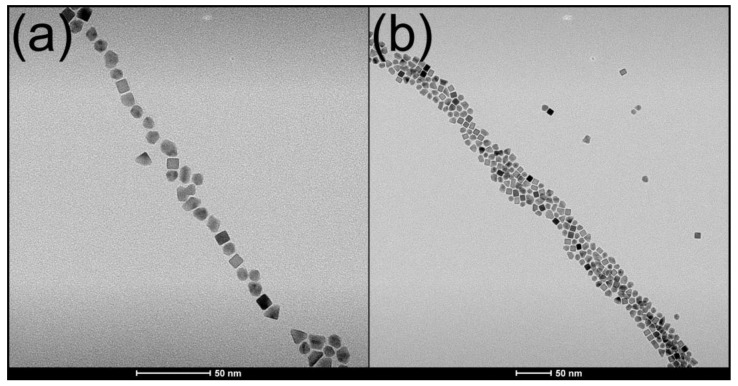
Transmission electron micrographs of dispersible platinum nanoparticles: (**a**) image of 8 nm cubic Pt NPs displaying {100}-faceted surfaces; (**b**) smaller, octahedral Pt NPs with dominant {111} facets can also be seen.

Microwave reactions in toluene with the Pt(acac)_2_ precursor OAm as a stabilizing agent, and ascorbic acid as a mild reducing agent, readily produced highly dispersible cubic Pt NPs with an average diameter of 8 nm (Figure 4). Microwave synthesis was performed using a Milestone StartSYNTH Microwave Synthesis Labstation (Milestone Srl, Italy) equipped with a single magnetron generator with a rotating diffuser operating at 2.45 GHZ, and a microprocessor-controlled power output of up to 1200 W. Temperature control was achieved by the instrument based on input from a contactless internal infrared sensor. A Weflon^TM^ button acted as a MW susceptor in the non-polar reaction mixture, which was heated under an applied power of 1200 W at 3.5 °C/minute to 135 °C and held for 30 min. Constant magnetic stirring was maintained throughout the reaction. After cooling to room temperature, dark brown Pt NPs were isolated from solution by several cycles of centrifugation and redispersal in ethanol. The OAm-capped Pt NPs (yield ~200 mg) were dispersed in 10 mL of toluene and stored as a colloidal solution that remained stable for a time period of over 1 year [7].

Decreasing reaction time and temperature to 30 min and 120 °C resulted in the formation of polycrystalline, irregularly shaped seed-like Pt NPs with an average diameter of 2–4 nm, which were used to functionalize the outer surfaces of photocatalytic nanocomposites. The reduction in particle size and irregular morphology was caused by insufficient energy in the reaction system to promote rapid precursor reduction and growth of faceted Pt NPs [43].

## 6. Nanopeapods

### 6.1. Hexaniobate Nanopeapods

Composite nanostructures that combine nanoscale sheets or scrolls of wide-bandgap semiconductors with metal or metal oxide nanoparticles have been the subject of much recent research for applications in photocatalysis and energy production and storage [53,54,55]. Scrolled nanoscale semiconductors feature hollow cores and greatly increased surface areas that invite diverse and extensive functionalization for catalysis applications [56,57,58,59,60]. The wide-bandgap semiconductor H_x_K_4−x_Nb_6_O_17_ (“hexaniobate”) is chemically desirable for its stability and unique structural features that can be used to produce exfoliated and scrolled morphologies [61,62]. Hexaniobate is of catalytic interest for its efficiency as an active generator and transport mediator of photoexcited charge carriers under UV irradiation [53,54,55]. Due to its wide bandgap, hexaniobate is often coupled with sensitizing dyes, metal or metal oxide nanoparticles, and other smaller-bandgap materials to make stable, efficient composite nanostructures for visible-range photocatalysis.

Nanopeapods (NPPs) are a unique type of composite nanoarchitecture in which nanoparticles are captured by scrolling layers of exfoliated nanosheets to form linear assemblies in the hollow inner core [61,62,63,64,65,66]. NPPs offer advanced, precise techniques for engineering the exfoliation, scrolling, and assembly processes, and represent a promising alternative for the placement of the catalytic, magnetic, or other types of NPs [63]. While nanopeapods can be produced via several synthetic routes utilizing a wide variety of nanoparticle and nanosheet components, one particularly versatile approach involves the encapsulation of dispersible NPs into ordered one-dimensional arrays inside scrolled hexaniobate nanosheets.

We have previously reported on the synthesis of hexaniobate-based NPPs incorporating nanoparticles such as Ag, Au, and CeO_2_, which showed interesting optical properties and feature materials that are relevant to catalytic applications [64,65,66]. Chen et al. recently reported on the formation of Au@Nb@H_x_K_1−x_NbO_3_ NPPs that displayed high catalytic activity for photoelectrochemical water splitting over a broad spectral range under an applied external voltage. The NPPs configuration of encapsulated linear particle arrays was found to promote plasmon–plasmon coupling between the catalyst NPs, which acted as “nanoantennas” to enable visible and NIR light harvesting by the UV-active niobate semiconductor [67]. For NPs that do not undergo self-assembly, templated growth methods can be used to create linear arrays for encapsulation into NPPs. Liu et al. reported a dual synthetic approach combining templated growth with high-temperature solid state reactions to encapsulate linear Pt chains with controlled interparticle separation into CoAl_2_O_4_ nanoshells. Controlling the placement and interaction of optically active NP arrays would drive the development of NPPs with tunable optical properties for applications in plasmonic waveguides and other 1D nano-optical devices [68]. Recent reports on templated growth describe more facile synthetic routes and increased yields [69,70].

As the main route for producing hexaniobate NPPs, solvothermal methods face significant challenges with respect to optimizing product yield, achieving precise control over pea and pod dimensions, increasing NP filling, and achieving complete hexaniobate exfoliation. Additionally, encapsulation of Pt NPs to form NPPs with good morphology and loading could not be achieved via conventional solvothermal synthesis.

### 6.2. MW Synthesis of Platinum@Hexaniobate Nanopeapods

**Figure 5:**
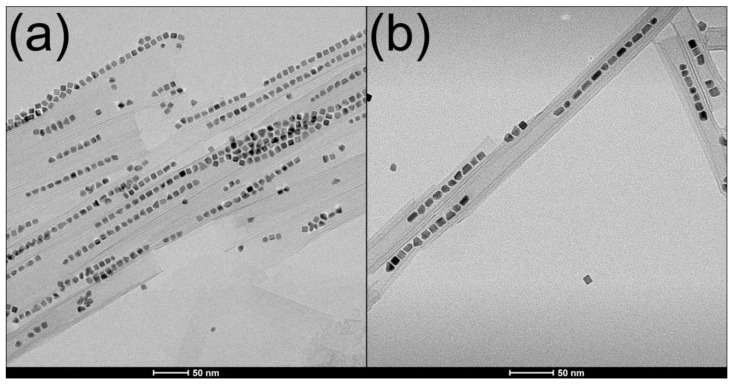
MW-synthesized Pt@HNB NPPs showing good morphology and loading. (**a**) Continuous one-dimensional assemblies of MW-synthesized 8-nm Pt nanocubes are encapsulated within scrolled hexaniobate layers; (**b**) demonstration of consistent interparticle spacing within encapsulated NP arrays.

We recently reported the formation of the new nanocomposite platinum@hexaniobate nanopeapods (Pt@HNB NPPs) in high yields via facile MW synthesis. Pt@HNB NPPs were selectively engineered as a heterogeneous hydrogen generation photocatalyst, and consist of linear Pt nanocube arrays within scrolled hexaniobate nanosheets. The MW synthetic route was adapted from the conventionally heated solvothermal method used by our group to produce Fe_3_O_4_, Ce, and Ag@HNB NPPs [61,65,66]. Crystalline hexaniobate was combined with the exfoliating agent tetrabutylammonium hydroxide (TBAOH) and surfactant OAm in toluene under magnetic stirring at room temperature. Then, 100 mg OAm-capped Pt nanocubes (synthesized via MW method described above) were added to the reaction mixture as a colloidal toluene suspension, followed by a Weflon^TM^ MW susceptor button and PTFE stir bar so that constant stirring at 650 rpm could be maintained throughout the reaction. The glass reaction vessel was heated to 135 °C via MW irradiation with an applied power of 1200 W and a ramp rate of 3.5 °C/min., then held for 105 min. After cooling to room temperature, the silvery-brown product was washed and isolated via 3–6 repetitions of centrifugation and redispersal. To isolate the peapods and remove any residual free Pt NPs, the product was redispersed in 25 mL hexane and centrifuged further for 1 min. The Pt@HNB NPPs (yield ~200 mg) were dispersed in hexane to form a stable colloidal suspension (Figure 5) [6].

### 6.3. Formation and Morphology of Platinum@Hexaniobate Nanopeapods

**Figure 6:**
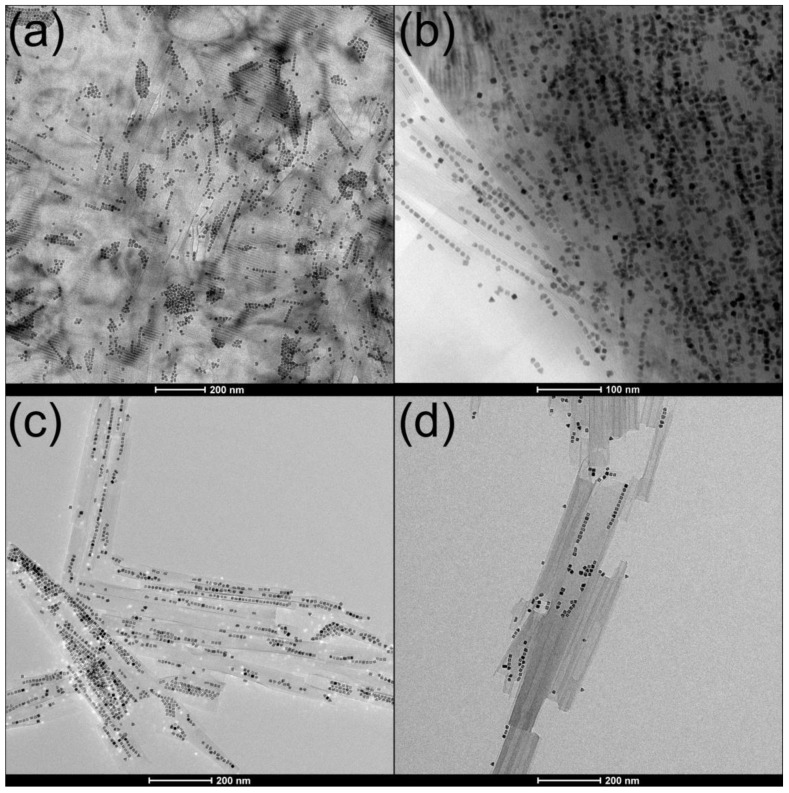
MW-synthesized Pt@HNB NPPs showing deviations from ideal morphology and loading: (**a**) extensive pre-organized NP assemblies on unscrolled hexaniobate surfaces that probably prevented NPP formation; (**b**) failure of NPPs to detach from bulk hexaniobate crystalline; (**c**) one- and two-dimensional NP arrays interfering with complete encapsulation; (**d**) bi-directional scrolling rather than complete NPP formation.

Control experiments to determine the optimal MW reaction conditions for morphologically uniform Pt@HNB NPPs with high rates of NSc conversion and NP loading were performed by systematically varying reaction time and temperature. This systematic variation produced a variety of effects that were observed in TEM images of the Pt@HNB NPPs, which provided an opportunity to investigate the formation mechanism. These images support a 3-step mechanism of “alignment → encapsulation → detachment”, while images of Pt@HNB NPPs synthesized under non-optimal conditions suggest that imbalances or deviations at each point of the process can hinder or even prevent the formation of NPPs. According to the proposed 3-step formation mechanism, delamination of the hexaniobate crystallite occurs as exfoliated nanosheets begin to break at certain crystallographic planes and convolve into nanoscrolls. Pre-aligned nanoparticles are captured by the scrolling process and subsequently encapsulated within the peapod before it detaches from the bulk hexaniobate (Figure 6a,b). The exact size and morphology of the NPP depends on structural defects in the nanosheets, rate of scrolling, nanoparticle size and alignment geometry, and reaction factors such as time and temperature.

In this study, optimal conditions for Pt@HNB NPPs with high NP loading and good morphology were found to be a reaction temperature of 135 °C and reaction time (including ramp) of 1 h and 45 min. More precise control over the concentration of Pt NPs and their interaction with the hexaniobate was also necessary for optimizing the Pt@HNB NPP synthesis protocol. Due to its flexibility and precision, the use of MW heating has been crucial to the successful formation of Pt@HNB NPPs. Typical MW yields range from 100–200 mg NPPs per synthesis, which is a significant improvement over the 10–50 mg produced by other methods. The quick reaction times, high yields, and reproducibility of both Pt NPs and Pt@HNB NPPs suggest that the MW heating method is ideal for large-scale production of high-quality platinum nanocomposites.

Results from the MW synthesis of Pt@HNB NPPs indicate that high temperatures can induce rapid exfoliation of hexaniobate nanosheets such that nearby NPs are unable to strongly adhere to the sheets or align before detachment occurs. This produces poorly filled or empty scrolls and formations of agglomerated NPs on the scroll exteriors (Figure 6c). For MW synthesis, reaction temperatures above 145 °C should not be used due to product quality and safety concerns. At more optimal temperatures (around 130 °C for MW), scroll formation is favored due to the rapid exfoliation that can occur under those conditions. Free NPs are readily captured within the scrolls and do not adhere as strongly to the outer surface.

Encapsulation selectivity by Pt@HNB NPPs for Pt NPs of different sizes and morphologies was studied via analysis of transmission electron micrographs. Suspensions containing both Pt nanocubes (5–15 nm) and smaller, irregular Pt NPs (2–4 nm) were utilized to synthesize Pt@HNB NPPs under optimal conditions. TEM images of these Pt@HNB NPPs showed that the larger Pt nanocubes were heavily favored over the smaller, irregular Pt NPs for encapsulation into the interior cavity, with successfully encapsulated Pt nanocubes having an average diameter of 7.8 nm (Figure 6d). Although a few very large (~13 nm) and very small (~5 nm) Pt NPs were seen to be encapsulated, the majority of encapsulated NPs (80%) were close to the measured average. It was found that NPs with cubic morphologies and narrow size distributions produced Pt@HNB NPPs with the highest filling rates and overall product yields. It is likely that properties associated with cubic nanoparticle morphology, such as the tendency to form self-assembled linear arrays on the bulk crystallite surface, were important factors in the formation of Pt@HNB NPPs with good morphology and high filling rates.

## 7. Conclusions

Typical synthesis methods for producing highly crystalline, dispersible Pt NPs present challenges in the form of reproducibility and accessibility. Platinum precursors are extremely dependent on having homogeneous energy and precursor dispersion throughout the reaction solution. Microwave heating has proved to be useful for providing that environment with a high degree of control over reaction rate and real-time monitoring of equilibrium temperature. Because it is not necessary to externally induce nucleation when using microwave heating methods, the dangerous reagent Fe(CO)_5_ was eliminated from the procedure, creating a more facile strategy with fewer health and environmental risks. This method can promote larger-scale production of high-quality Pt NPs for photocatalytic applications.

MW heating was also used to produce high-quality intricate nanocomposites in the form of tightly scrolled, morphologically uniform, highly loaded Pt@HNB NPPs. This facile and readily reproducible method produces much higher yields (200 mg vs. 10–20 mg per synthesis) compared to solvothermal and solvent evaporation methods. Pt@HNB NPPs were developed with a specific purpose in mind: photocatalytic water splitting to generate H_2_ under visible light irradiation. This unique nanopeapods architecture offers excellent potential for improving the efficiency of photocatalytic water splitting by heterogeneous catalysts [15].

## Figures and Tables

**Table 1:** Advantages of MW irradiation over conventional heating routes.

Usage/Reaction	Benefits of MW Heating
General operation	Risk reduction (health and safety) [2]
	Automation [9] Scalability (experimental→batch) [10]
Experimental parameters	Mild reaction conditions [1] Reduced reaction times [4]
	Precise temperature control [11] In-situ temperature and pressure monitoring [12] Lack of thermal gradients [8]
Colloidal catalyst NPs	Smaller size distribution [13]
	Spontaneous nucleation event [14]
	Homogeneous nucleation and growth [14]
	Prevention of agglomeration [7]
Catalyst nanocomposites	Intricate nanocomposite production [15] Monodispersed products [16] Elucidation of formation mechanisms [6]
	Repeatability [17] Uniform heat distribution improves product yields [18]

**Table 2:** Summary of loss tangent (tan δ) values for solvents commonly used in MW synthesis.

LOW: tan δ < 0.1		MEDIUM: 0.1 ≤ tan δ ≤ 0.5		HIGH: tan δ > 0.5	
Solvent	tan δ	Solvent	tan δ	Solvent	tan δ
Acetonitrile	0.062	DMF	0.161	1,2-Ethanediol	1.350
THF	0.047	1,2-Dichloroethane	0.127	Ethanol	0.941
Toluene	0.040	Water	0.123	DMSO	0.659

## References

[1] Kappe C.O., Dallinger D. (2009). Controlled microwave heating in modern organic synthesis: Highlights from the 2004–2008 literature. Mol. Divers..

[2] Hayes B.L. (2002). Microwave Synthesis-Chemistry at the Speed of Light.

[3] Bilecka I., Niederberger M. (2010). Microwave chemistry for inorganic nanomaterials synthesis. Nanoscale.

[4] Kappe C.O. (2002). High-speed combinatorial synthesis utilizing microwave irradiation. Curr. Opin. Chem. Biol..

[5] Kappe C.O. (2008). Microwave dielectric heating in synthetic organic chemistry. Chem. Soc. Rev..

[6] Davis-Wheeler Chin C. (2018). Platinum@hexaniobate Nanopeapods: Sensitized Composite Architectures for Photocatalytic Hydrogen Evolution under Visible Light Irradiation.

[7] Davis-Wheeler Chin C., Akbarian-Tefaghi S., Reconco-Ramirez J., Wiley J.B. (2018). Rapid microwave synthesis and optical activity of highly crystalline platinum nanocubes. MRS Commun..

[8] Baghbanzadeh M., Carbone L., Cozzoli P.D., Kappe C.O. (2011). Microwave-Assisted Synthesis of Colloidal Inorganic Nanocrystals. Angew. Chem. Int. Ed..

[9] Gawande M.B., Shelke S.N., Zboril R., Varma R.S. (2014). Microwave-Assisted Chemistry: Synthetic Applications for Rapid Assembly of Nanomaterials and Organics. Acc. Chem. Res..

[10] Goyal H., Mehdad A., Lobo R.F., Stefanidis G., Vlachos D.G. (2019). Scaleup of a Single-Mode Microwave Reactor. Ind. Eng. Chem. Res..

[11] Kappe C.O. (2013). ChemInform Abstract: How to Measure Reaction Temperature in Microwave-Heated Transformations. Chem. Soc. Rev..

[12] Hayden S., Damm M., Kappe C.O. (2012). On the Importance of Accurate Internal Temperature Measurements in the Microwave Dielectric Heating of Viscous Systems and Polymer Synthesis. Macromol. Chem. Phys..

[13] Carbone L. (2013). Wet-Chemical Synthesis Techniques for Colloidal Plasmonic Nanostructures Assisted by Convective or Microwave Dielectric Heating. Handbook of Molecular Plasmonics.

[14] Carbone L., Cozzoli P.D. (2010). Colloidal heterostructured nanocrystals: Synthesis and growth mechanisms. Nano Today.

[15] Davis-Wheeler Chin C., Fontenot P.R., Rostamzadeh T., Treadwell L.J., Schmehl R.H., Wiley J.B. (2021). Platinum@Hexaniobate Nanopeapods: A Directed Catalytic Nanocomposite Architecture for Sensitized H2 Production Under Visible Light Irradiation.

[16] Horikoshi S., Serpone N. (2013). Nanoparticle Synthesis through Microwave Heating. Microwaves in Nanoparticle Synthesis.

[17] Zhu Y.-J., Chen F. (2014). Microwave-Assisted Preparation of Inorganic Nanostructures in Liquid Phase. Chem. Rev..

[18] Deshayes S., Liagre M., Loupy A., Luche J.-L., Petit A. (1999). Microwave activation in phase transfer catalysis. Tetrahedron.

[19] Kitchen H.J., Vallance S.R., Kennedy J.L., Ruiz N.T., Carassiti L., Harrison A., Whittaker A.G., Drysdale T.D., Kingman S., Gregory D.H. (2014). Modern Microwave Methods in Solid-State Inorganic Materials Chemistry: From Fundamentals to Manufacturing. Chem. Rev..

[20] Nishioka M., Miyakawa M., Daino Y., Kataoka H., Koda H., Sato K., Suzuki T.M. (2013). Single-Mode Microwave Reactor Used for Continuous Flow Reactions under Elevated Pressure. Ind. Eng. Chem. Res..

[21] Sauks J.M., Mallik D., Lawryshyn Y., Bender T., Organ M. (2013). A Continuous-Flow Microwave Reactor for Conducting High-Temperature and High-Pressure Chemical Reactions. Org. Process. Res. Dev..

[22] CEM Discover 2.0. https://cem.com/en/discover-2.

[23] Biotage Biotage® Initiator + SP Wave. https://www.biotage.com/spwave-peptide-synthesis-0.

[24] Anton-Paar MonowaveTM 400/200. https://www.anton-paar.com/corp-en/products/details/microwave-synthesis-monowave-400200/.

[25] Sturm G.S., Verweij M.D., Stankiewicz A.I., Stefanidis G. (2014). Microwaves and microreactors: Design challenges and remedies. Chem. Eng. J..

[26] Microwave Research & Applications BP-210. https://www.microwaveresearch.com/BP-210.php.

[27] CEM Liberty Blue. https://cem.com/liberty-blue/.

[28] CEM MARS6. https://cem.com/en/mars-6-synthesis.

[29] (2007). Milestone Segmented Rotor and Fiber Optic Probes.

[30] Robinson J., Kingman S., Irvine D., Licence P., Smith A., Dimitrakis G., Obermayer D., Kappe C.O. (2010). Understanding microwave heating effects in single mode type cavities—theory and experiment. Phys. Chem. Chem. Phys..

[31] Cushing B.L., Kolesnichenko V., O’Connor C.J. (2004). Recent Advances in the Liquid-Phase Syntheses of Inorganic Nanoparticles. Chem. Rev..

[32] Leong G.J., Schulze M.C., Strand M.B., Maloney D., Frisco S.L., Dinh H.N., Pivovar B., Richards R.M. (2014). Shape-directed platinum nanoparticle synthesis: Nanoscale design of novel catalysts: A review of shape-directed platinum nanoparticle synthesis. Appl. Organomet. Chem..

[33] Casavola M., Buonsanti R., Caputo G., Cozzoli P.D. (2008). Colloidal Strategies for Preparing Oxide-Based Hybrid Nanocrystals. Eur. J. Inorg. Chem..

[34] Pinna N., Niederberger M. (2008). Surfactant-Free Nonaqueous Synthesis of Metal Oxide Nanostructures. Angew. Chem. Int. Ed..

[35] Cozzoli P.D., Pellegrino T., Manna L. (2006). Synthesis, properties and perspectives of hybrid nanocrystal structures. Chem. Soc. Rev..

[36] Xia Y., Xiong Y., Lim B., Skrabalak S.E. (2008). Shape-Controlled Synthesis of Metal Nanocrystals: Simple Chemistry Meets Complex Physics?. Angew. Chem. Int. Ed..

[37] Niederberger M., Pinna N. (2009). Metal Oxide Nanoparticles in Organic Solvents; Engineering Materials and Processes.

[38] Bönnemann H., Khelashvili G., Bönnemann H. (2010). Efficient fuel cell catalysts emerging from organometallic chemistry. Appl. Organomet. Chem..

[39] Mazumder V., Sun S. (2009). Oleylamine-Mediated Synthesis of Pd Nanoparticles for Catalytic Formic Acid Oxidation. J. Am. Chem. Soc..

[40] Peng Z., Yang H. (2009). Designer platinum nanoparticles: Control of shape, composition in alloy, nanostructure and electrocatalytic property. Nano Today.

[41] Yu W., Tu A.W., Liu H. (1999). Synthesis of Nanoscale Platinum Colloids by Microwave Dielectric Heating. Langmuir.

[42] Carpenter M.K., Moylan T.E., Kukreja R.S., Atwan M.H., Tessema M.M. (2012). Solvothermal Synthesis of Platinum Alloy Nanoparticles for Oxygen Reduction Electrocatalysis. J. Am. Chem. Soc..

[43] Long N.V., Chien N.D., Hayakawa T., Hirata H., Lakshminarayana G., Nogami M., Gandham L. (2009). The synthesis and characterization of platinum nanoparticles: A method of controlling the size and morphology. Nanotechnol..

[44] Kang Y., Li M., Cai Y., Cargnello M., Diaz R.E., Gordon T.R., Wieder N.L., Adzic R.R., Gorte R.J., Stach E.A. (2013). Heterogeneous Catalysts Need Not Be so “Heterogeneous”: Monodisperse Pt Nanocrystals by Combining Shape-Controlled Synthesis and Purification by Colloidal Recrystallization. J. Am. Chem. Soc..

[45] Wang C., Daimon H., Lee Y., Kim A.J., Sun S. (2007). Synthesis of Monodisperse Pt Nanocubes and Their Enhanced Catalysis for Oxygen Reduction. J. Am. Chem. Soc..

[46] Sanz J.M., Ortiz D., De La Osa R.A., González F., Brown A.S., Losurdo M., Everitt H.O., Moreno F. (2013). UV Plasmonic Behavior of Various Metal Nanoparticles in the Near- and Far-Field Regimes: Geometry and Substrate Effects. J. Phys. Chem. C.

[47] Wang C., Daimon H., Onodera T., Koda T., Sun S. (2008). A General Approach to the Size- and Shape-Controlled Synthesis of Platinum Nanoparticles and Their Catalytic Reduction of Oxygen. Angew. Chem. Int. Ed..

[48] Chen J., Lim B., Lee E.P., Xia Y. (2009). Shape-controlled synthesis of platinum nanocrystals for catalytic and electrocatalytic applications. Nano Today.

[49] Dahal N., García S., Zhou J., Humphrey S.M. (2012). Beneficial Effects of Microwave-Assisted Heating versus Conventional Heating in Noble Metal Nanoparticle Synthesis. ACS Nano.

[50] Kou J., Bennett-Stamper C., Varma R.S. (2013). Green Synthesis of Noble Nanometals (Au, Pt, Pd) Using Glycerol under Microwave Irradiation Conditions. ACS Sustain. Chem. Eng..

[51] Komarneni S., Li D., Newalkar B., Katsuki A.H., Bhalla A.S. (2002). Microwave—Polyol Process for Pt and Ag Nanoparticles. Langmuir.

[52] Wang Y., Ren J., Deng K., Gui A.L., Tang Y. (2000). Preparation of Tractable Platinum, Rhodium, and Ruthenium Nanoclusters with Small Particle Size in Organic Media. Chem. Mater..

[53] Cao Y., Jiang L., Guo H., Zheng Q. (2014). Nano-layered K_4_Nb_6_O_17_ as an efficient photocatalyst for methyl orange degradation: Influence of solution pH and surface-dispersed gold nanoparticles. J. Mol. Catal. A Chem..

[54] Maeda K., Eguchi M., Youngblood W.J., Mallouk T. (2008). Niobium Oxide Nanoscrolls as Building Blocks for Dye-Sensitized Hydrogen Production from Water under Visible Light Irradiation. Chem. Mater..

[55] Sarahan M.C., Carroll E.C., Allen M., Larsen D.S., Browning N.D., Osterloh F.E. (2008). K_4_Nb_6_O_17_-derived photocatalysts for hydrogen evolution from water: Nanoscrolls versus nanosheets. J. Solid State Chem..

[56] Domen K., Yoshimura J., Sekine T., Tanaka A., Onishi T. (1990). A novel series of photocatalysts with an ion-exchangeable layered structure of niobate. Catal. Lett..

[57] Maeda K., Sahara G., Eguchi M., Ishitani O. (2015). Hybrids of a Ruthenium (II) Polypyridyl Complex and a Metal Oxide Nanosheet for Dye-Sensitized Hydrogen Evolution with Visible Light: Effects of the Energy Structure on Photocatalytic Activity. ACS Catal..

[58] Osterloh F. (2008). Inorganic Materials as Catalysts for Photochemical Splitting of Water. Chem. Mater..

[59] Sarac F.E., Yilmaz C., Acar F.Y., Unal U. (2012). CdTe quantum dot sensitized hexaniobate nanoscrolls and their photoelectrochemical properties. RSC Adv..

[60] Maeda K., Eguchi M., Lee S.-H.A., Youngblood W.J., Hata H., Mallouk T.E. (2009). Photocatalytic Hydrogen Evolution from Hexaniobate Nanoscrolls and Calcium Niobate Nanosheets Sensitized by Ruthenium (II) Bipyridyl Complexes. J. Phys. Chem. C.

[61] Adireddy S., Carbo C.E., Yao Y., Vargas J.M., Spinu L., Wiley J.B. (2013). High-Yield Solvothermal Synthesis of Magnetic Peapod Nanocomposites via the Capture of Preformed Nanoparticles in Scrolled Nanosheets. Chem. Mater..

[62] Rostamzadeh T., Adireddy S., Wiley J.B. (2015). Formation of Scrolled Silver Vanadate Nanopeapods by Both Capture and Insertion Strategies. Chem. Mater..

[63] Yao Y., Chaubey G.S., Wiley J.B. (2011). Fabrication of Nanopeapods: Scrolling of Niobate Nanosheets for Magnetic Nanoparticle Chain Encapsulation. J. Am. Chem. Soc..

[64] Adireddy S., Carbo C.E., Rostamzadeh T., Vargas J.M., Spinu L., Wiley J.B. (2014). Peapod-Type Nanocomposites through the In Situ Growth of Gold Nanoparticles within Preformed Hexaniobate Nanoscrolls. Angew. Chem. Int. Ed..

[65] Adireddy S., Rostamzadeh T., Carbo C.E., Wiley J.B. (2014). Particle Placement and Sheet Topological Control in the Fabrication of Ag–Hexaniobate Nanocomposites. Langmuir.

[66] Rostamzadeh T., Adireddy S., Davis-Wheeler Chin C., Abdallah M.H., Wiley J.B. (2019). Formation of Mixed-Metal Ceria Nanopeapod Composites within Scrolled Hexaniobate Nanosheets. ChemNanoMat.

[67] Chen Y.C., Hsu Y.K., Popescu R., Gerthsen D., Lin Y.G., Feldmann C. (2018). Au@Nb@H_x_K_1-x_NbO_3_ nanopeapods with near-infrared active plasmonic hot-electron injection for water splitting. Nat. Commun..

[68] Liu L., Lee W., Scholz R., Pippel E., Gösele U. (2008). Tailor-Made Inorganic Nanopeapods: Structural Design of Linear Noble Metal Nanoparticle Chains. Angew. Chem. Int. Ed..

[69] Byoun W., Yoo H. (2017). Peapod Assemblies of Au and Au/Pt Nanoparticles Encapsulated within Hollow Silica Nanotubes. ChemistrySelect.

[70] Takai A., Sakamoto Y., Terasaki O., Yamauchi Y., Kuroda K. (2013). Platinum Nanopeapods: Spatial Control of Mesopore Arrangements by Utilizing a Physically Confined Space. Chem. A Eur. J..

